# Antimicrobial use in Chinese swine and broiler poultry production

**DOI:** 10.1186/s13756-015-0050-y

**Published:** 2015-04-28

**Authors:** Vikram Krishnasamy, Joachim Otte, Ellen Silbergeld

**Affiliations:** Johns Hopkins University Bloomberg School of Public Health, 615 N. Wolfe Street, Room WB602, Baltimore, MD 21205 USA; Food and Agriculture Organization, Room C-510, Viale delle Terme di Caracalla, Rome, 00153 Italy; Johns Hopkins University Bloomberg School of Public Health, 615 N. Wolfe Street, Room E6644, Baltimore, MD 21205 USA

**Keywords:** Agriculture, Anti-bacterial agents, Animals, China, Microbial drug resistance, Poultry, Swine, Food supply

## Abstract

**Background:**

Antimicrobial use for growth promotion in food animal production is now widespread. A major concern is the rise of antimicrobial resistance and the subsequent impact on human health. The antimicrobials of concern are used in concentrated animal feeding operations (CAFOs) which are responsible for almost all meat production including swine and poultry in the US. With global meat consumption rising, the CAFO model has been adopted elsewhere to meet this demand. One such country where this has occurred is China, and evidence suggests 70% of poultry production now occurs outside of traditional small farms. Moreover, China is now the largest aggregate consumer of meat products in the world. With this rapid rise in consumption, the Chinese production model has changed along with the use of antimicrobials in feeds. However, the specific antibiotic use in the Chinese food animal production sector is unclear. Additionally, we are aware of high quantities of antimicrobial use because of reports of high concentrations of antimicrobials in animal waste and surface waters surrounding animal feeding operations.

**Methods:**

In this report, we estimate the volume of antibiotics used for swine and poultry production as these are the two meat sources with the highest levels of production and consumption in China. We adopt a model developed by Mellon et al. in the US for estimating drug use in feed for poultry and swine production to estimate overall antimicrobial use as well as antimicrobial use by class.

**Results:**

We calculate that 38.5 million kg [84.9 million lbs] were used in 2012 in China’s production of swine and poultry. By antibiotic class, the highest weights are tetracyclines in swine and coccidiostats in poultry.

**Conclusions:**

The volume of antimicrobial use is alarming. Although there are limitations to these data, we hope our report will stimulate further analysis and a sense of urgency in assessing the consequences of such high levels of utilization in terms of antibiotic resistance in the food supply and the environment.

## Background

The use of antimicrobials in food animal production is now well known in the US and many other countries [1]. Antimicrobial use in feeds for food animal production first started in the 1940s when they were added to feeds used in broiler poultry production [2]. It was claimed that chickens gained more weight in a shorter amount of time resulting in greater feed efficiency [3,4]. In 1925, a 1.13 kg [2.5 lb] chicken could be produced in 112 days. By 1950, this had been cut to 70 days. As of 2010, a chicken weighing greater than 2.27 kg [5 lbs] can be produced in less than 50 days [5]. The practice has since been adopted in the production of other food animals in the United States, throughout the developed world, and in lesser-developed countries such as China and Brazil [6,7]. It remains unclear why antimicrobials cause food animals to gain weight more quickly. Some hypothesize that they lead to decreased illness allowing weight to accumulate faster [8,9]. However, no convincing evidence has been produced to support this idea [10].

Prior to the rise in antimicrobial use came a change in how food animals were produced. Small farms were rapidly replaced by operations with much higher densities of animals. In 1950, chickens consumed in the US came from more than 1.5 million farms across the country with about 355 chickens per farm [5]. By 2012, there were about 16,000 farms that produced less than 2,000 broilers per year [11]. In addition, by 2012, there were over 15,000 operations that produced greater than 100,000 broilers annually [11]. These trends were repeated in swine production [11,12]. Some have attributed lower consumer meat prices to this rise in larger operations [13]. Additionally, preceding this rise of animal feeding operations was a trend towards vertical integration whereby corporations controlled the production chain [14]. Many farmers now raise animals under contract with these corporations and do not own the animals in their operations [13]. This has allowed vertically integrated corporations to dictate terms for each operation [13]. The largest poultry integrators now produce over 500,000 chickens annually, with some corporations controlling thousands of operations [15].

This rise in poultry and swine production has paralleled and driven increases in demand by consumers in the United States. Since 1950, per capita meat consumption in the United States has risen dramatically. One study noted US per capita meat consumption doubled between 1909 and 2007 [16]. Consumption in the European Union also doubled more recently between 1961 and 2003 [16]. Additionally, developing countries have seen marked consumption increases. The World Health Organization notes that consumption in developing countries more than doubled in the second half of the 20^th^ century [17]. It is estimated that annual worldwide meat production will be 376 million metric tons [414 million short tons] by 2030, up from 218 million metric tons [240 million short tons] in 1999 [17]. Increasing urbanization, incomes, and populations are significant factors in this trend [7]. While developing country consumption still lags behind developed countries, consumption is rising more rapidly than in developed countries [17-19].

**Figure 1:**
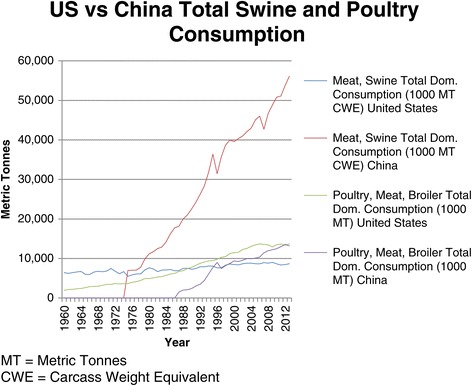
US and China swine and poultry consumption 1960-2013. Source: USDA Foreign Agricultural Service. MT = Metric Tonnes; CWE = Carcass Weight Equivalent.

One such country where production and consumption have both increased rapidly is China. Total meat consumption in China is higher than any other country [19]. Total and per capita pork consumption is the highest in the world making pork the most popular meat in China [20]. As of 2012, the Chinese consumed 38.1 kg [84 lbs] of pork per person annually while Americans consumed 26.8 kg [59 lbs] per person [21]. In the 1970s, total meat consumption was only one-third of the United States [21]. China surpassed the US in total meat consumption in 1992. Since then, demand has only risen [18,21]. Currently, half of the world’s living pigs reside in China, more than 470 million. Moreover, in 2011, 660 million swine were produced in China [18]. With rapid urbanization and increasing incomes, we have seen meat consumption continue to increase [18,20,22]. And, pork it seems, will remain the most popular meat for the time being [23]. Figure 1 notes the trends in total swine and poultry consumption in the United States and China from 1960 to 2013 [24].

A shift to larger operations has supported increases in Chinese food animal production. In 1998, 30% of broilers in China were produced on farms that produced greater than 2000 broilers annually. In 2009, this number was 70% [7,25]. Between 2007 and 2009, the number of poultry operations in China with more than 100,000 broilers increased by 34% [25]. In 2014, this number is likely to be higher although we do not yet have data to confirm this. Similar rapid intensification has occurred in the pork industry [20]. In 2000, it was estimated that 74% of swine production occurred on backyard farms with an annual production of 1-49 heads. Commercial farms that produced greater than 1000 heads per year accounted for only 5% of swine production. By 2010, these commercial farms accounted for 12% while backyard farms had decreased to 37% [20,22].

A direct comparison of swine production in the US and China is provided in Table 1 [18]. Between the years 2000 and 2012, there were increases in the number of swine slaughtered in both the US and China. Pig inventories also increased. While the number of swine slaughtered is much larger in China than in the US, carcass weight is still greater in the US. Table 2 provides a comparison between the US and Chinese poultry production industries between the years 2000 and 2012. Again, both countries experienced increases in production. Additionally, carcass weights remain higher in the US than in China.

**Table 1:** **Chinese and US swine industry comparisons**

	**2000**	**2012**	**Change (%)**
*China*			
Pig inventory	388,298,000	465,500,000	19.9
No slaughtered	468,796,366	670,949,440	43.1
Carcass weight (kg)	76.5	73.2	-4.3
Production (tonnes)	35,862,922	49,113,499	36.9
Offtake rate	1.21	1.44	19.0
Prod (kg)/Invent	92.36	105.51	14.2
*USA*			
Pig inventory	59,342,000	66,412,800	11.9
No slaughtered	93,815,987	107,497,830	14.6
Carcass weight (kg)	89.4	92.3	3.2
Production (tonness)	8,386,510	9,921,970	18.3
Offtake rate	1.58	1.62	2.5
Prod (kg)/Invent	141.33	149.40	5.7
*China/USA (%)*			
Carcass weight	85.6	79.3	-7.3
Offtake rate	76.6	88.9	16.1
Prod (kg)/invent	65.4	70.6	8.1

Source: FAOSTAT [18].

**Table 2:** **Chinese and US poultry industry comparisons**

	**2000**	**2012**	**Change (%)**
*China*			
Poultry Inventory*	3,500,000,000	5,300,000,000	51.4
Laying Hens	1,895,000,000	2,620,000,000	38.3
No slaughtered	6,237,038,000	9,178,430,000	47.2
Carcass weight (kg)	1.35	1.38	2.2
Production (tonnes)	8,426,862	12,667,151	50.3
Offtake rate	1.78	1.73	-2.8
Prod (kg)/Invent	2.41	2.39	-0.7
*USA*			
Poultry Inventory*	1,860,000,000	1,929,600,000	3.7
Laying Hens	328,300,000	339,698,000	3.5
No slaughtered	8,470,387,000	8,650,779,000	2.1
Carcass weight (kg)	1.65	1.97	19.6
Production (tonnes)	13,947,000	17,038,000	22.2
Offtake rate	4.55	4.48	-1.6
Prod (kg)/Invent	7.50	8.83	17.8
*China/USA (%)*			
Carcass weight	82.1	70.1	-14.6
Offtake rate	39.1	38.6	-1.3
Prod (kg)/invent	32.1	27.1	-15.7

Source: FAOSTAT [18].

*Poultry inventory includes laying hens.

As a result of rising production and the shift to larger animal feeding operations, there is a concern about the adverse consequences of these systems [20]. This is especially true in China where high consumption of meat among a population of 1.3 billion and the shift to intensive agricultural practices have led to environmental consequences [26-28]. Many argue in favor of accounting for the negative externalities resulting from this food animal production [5,6,13,15,29,30]. The risks of concentrated animal feeding operations are many and include driving the emergence and dissemination of antimicrobial resistance associated with the use of antimicrobials in animal feeds for growth promotion [13]. They also include groundwater and surface water contamination from the high quantities of animal waste produced. It is estimated that 500 million tons of solid waste are generated annually by animal feeding operations in the US [31]. A US Government Accountability Office report concluded that an 800,000 hog operation can generate more waste than the city of Philadelphia [32]. In China, it is estimated that 1.7 million metric tons [1.9 billion short tons] of animal waste were generated in 2010 [33].

One of the important aspects of intensive food animal production is the association with the use of antimicrobials in feeds. Antimicrobials are an essential component of clinical medicine that are threatened by increasing resistance to multiple drugs in major human pathogens. The reasons for increases in resistance are related to the overuse of these drugs in both clinical medicine and food animal production [34,35]. While there have been national and international programs to regulate clinical use of antimicrobials, only limited efforts have been made to reduce drug use in animal feeds [1,35,36]. Hence, regulation becomes an important mechanism to restrict use in feeds. In China, data indicate significant evidence of increasingly prevalent antibiotic resistance genes in the soils and wastewaters around Chinese livestock farms [37,38]. Currently, there are no official data on antibiotic use in Chinese agriculture. Some studies in the literature have noted a 2007 survey in which Chinese antimicrobial production was 210,000 metric tons [231,485 short tons] with 46% used in the livestock industry [37,39]. While this survey is reported in the literature in several studies, we have not been able to identify the survey and its methods.

In this paper, we seek to estimate the quantity of antimicrobials used in broiler poultry and swine feeds in Chinese food animal production. We use data on food animal antimicrobial utilization from the US assuming that Chinese practices are similar. This method does have shortcomings which we detail in the discussion. To our knowledge, there are no data or estimates of antimicrobial use in the poultry and swine production sectors in China.

## Methods

To estimate antimicrobial use, we adopted the methodology of Mellon et al. (2001) [40]. Their estimates are close to estimates published by the Food and Drug Administration (FDA) using industry reported data [41]. This methodology generated estimates of the volume of antimicrobials by weight used in US food animal production based on FDA registration data, recommended usage of feed formulations, and production levels. The formula used by Mellon et al. for poultry is as follows:

- Use = N x P x F x D where:
- N = Number of animals in the stage
- P = Percent of animals treated
- F = Feed consumed per animal in each stage
- D = Average dose of antimicrobials per pound of feed

Given that China has adopted US agricultural methods, we used this methodology for our estimates of antimicrobial use in animal feeds in China for both swine and poultry production [42]. For poultry, we specifically selected chicken while excluding ducks, geese, turkey, and quail. As discussed below, we estimate use in broiler chickens specifically.

It is also important to note that all results are by overall weight. Another method for calculating antimicrobial use involves calculating defined animal daily doses which are the average maintenance doses per day [43]. We adopted the methodology from Mellon et al. which was based on total weight.

### Poultry

We used data from the statistics division of the Food and Agriculture Organization (FAOSTAT) to identify the number of chickens slaughtered in 2012, the latest data available [18]. This number was approximately 9.2 billion chickens. We also accounted for the number of laying hens since, as they were likely to be included in the number of chickens slaughtered. For 2012, the total number of layers was about 2.6 billion. In order to adjust FAOSTAT data to only broiler chickens, we assumed that each laying hen lived for an average of 1.5 years before slaughter. We then assumed two-thirds of these laying hens were slaughtered in 2012. The International Egg Commission provides an alternate estimate of the number of laying hens in China: 800– 1,000 million [44]. We used the Food and Agriculture Organization (FAO) data because the use of FAO data is widespread in the literature. Mellon et al. provided details on six combinations of antimicrobials used in each stage of poultry production. Poultry are produced in a Starting stage and a Growing/Finishing stage. Mellon et al. generated information on the average concentration of each combination per pound of feed and assumed that feeds contained 80% of the maximum concentration dose allowed by the FDA. In their tables, they also indicated the percent of broilers given feeds with each combination of antimicrobials and the pounds of feed consumed per bird in each stage of growth. Antimicrobial use in feed was calculated separately for each growth stage.

### Swine

We used data from FAOSTAT to identify the number of swine slaughtered in China in 2012 which was approximately 671 million. Swine are also grown in several stages from birth to slaughter. We assumed about a 4% loss from starter to feeder phase and then assumed about a 2% loss from feeder to slaughter phase based on the Mellon et al. methodology. The number of swine in each stage is included in the tables below. The formula used by Mellon et al. is as follows:

- Use = N x F x T x C where:
- N = Number of animals in a group for the given stage
- F = Estimated feed consumed per day
- T = Duration in days in each phase that swine received antimicrobials in their feed
- D = Dose of antimicrobials in the feed

Similar to poultry, Mellon et al. provided details on the percent of swine treated with each antimicrobial combination in each phase of growth, the pounds per day of feed consumed in each stage, average days administered, and the concentration of each antimicrobial combination in feed. They assumed antimicrobials were not given for the whole period of each stage. They also assumed the concentrations of antimicrobials in feeds were 70-85% of the maximum concentration allowed. Antimicrobial use was calculated for Starter, Feeder, Finisher, and Breeder phases. Because we were unable to identify the number of breeding pigs in China, we used an estimate based on the ratio of breeding pigs to number of slaughtered pigs provided in Mellon et al.

## Results

### Poultry

For poultry, our estimates suggest that slightly more than 4.5 million kg [9.9 million lbs] of antimicrobials were used in production for 2012 (Table 3). In the Pre-starter and Starter phases, 25% of total antimicrobials were used. In the Grower and Finisher phases, 75% of total antimicrobials were used.

**Table 3:** **Poultry use by stage**

**Stage**	**Number of Broilers**	**Totals (g)**	**Total (kg)**	**Percentage**	**Total/1000 animals**
Pre-starter/Starter	7,431,763,333	1,148,764,817	1,148,765	25%	0.155
Grower/Finsher	7,431,763,333	3,404,633,473	3,404,633	75%	0.458
Overall	7,431,763,333	4,553,398,290	4,553,398	100%	

A breakdown per combination of antimicrobials used is provided in Table 4. For the Pre-starter and Starter phases, antimicrobial combinations 1, 2, and 6 are used in the highest percentage of broilers, 25%. Combination 2 results in the greatest overall weight used; 301,488 kg [664,667 lbs]. For the Grower and Finisher phases, combinations 1 and 6 are used in the highest percentage of broilers at 28%. Combination 1 results in the highest overall weight used; 796,067 kg [1,755,027 lbs].

**Table 4:** **Breakdown by poultry antimicrobial combination**

**Pre-starter and Starter Phases**					
**Antimicrobial (AM) combination**	**Number of chickens**	**Percent of birds treated**	**Pounds of Feed**	**g AM/lb of feed**	**Total in kg**
1. bambermycin, amprolium, ethopabate, roxarsone	7,431,763,333	25%	2.25	0.061680	257,845
2. BMD, roxarsone, monensin	7,431,763,333	25%	2.25	0.072120	301,488
3. chlortetracycline, roxarsone	7,431,763,333	5%	2.25	0.218160	182,398
4. penicillin, amprolium, ethopabate	7,431,763,333	5%	2.25	0.066840	55,883
5. lincomycin, roxarsone, amprolium, ethopabate	7,431,763,333	15%	2.25	0.066400	166,546
6. virginiamycin, roxarsone, salinomycin	7,431,763,333	25%	2.25	0.044160	184,605
Total					1,148,765
**Grower and Finisher Phases**					
**Antimicrobial combination**	**Number of chickens**	**Percent of birds treated**	**Pounds of feed**	**g AM/lb of feed**	**Total in kg**
1. bambermycin, lasalocid, roxarsone	7,431,763,333	28%	6	0.06376	796,067
2. erythromycin, arsanilic acid, zoalene	7,431,763,333	10%	6	0.1184	527,952
3. chlortetracycline, roxarsone, monensin	7,431,763,333	5%	6	0.26216	584,493
4. penicillin, roxarsone, zoalene	7,431,763,333	5%	6	0.08356	186,299
5. lincomycin, lasalocid, roxarsone	7,431,763,333	20%	6	0.06416	572,186
6. virginiamycin, monensin, roxarsone	7,431,763,333	28%	6	0.05908	737,635
Total					3,404,633

### Swine

The results from our estimates shown in Table 5 indicate that 34 million kg [75 million lbs] of antimicrobials were used in the production of swine in 2012. About 70% of the total antimicrobial use was during the finishing phase of swine production. A breakdown per combination of antimicrobials used is provided in Table 6. For the Starting phase, chlortetracycline alone was used in the highest percentage of swine, 50%. Combinations 1 and 2 along with chlortetracycline resulted in the largest weights of antibiotics used in this stage: 996,440 kg [2,196,774 lbs]. In the Feeding phase, bacitracin was used in 55% of all swine. Chlortetracycline again resulted in the greatest weight used: 1,638,378 kg [3,612,005 lbs]. For the Finishing phase, bacitracin again was the most commonly used antibiotic, 60% of all swine. Chlortetracycline alone once again resulted in the largest weight used: 6,886,692 kg [15,182,557 lbs]. Finally, for the Breeding phase, chlortetracycline was used in 85% of swine. As a result, chlortetracycline contributed the largest weight to the overall total in this phase: 171,338 kg [377,736 lbs].

**Table 5:** **Swine use by stage**

**Stage**	**Number of swine**	**Totals (g)**	**Total (kg)**	**Percentage**	**Total/1000 animals (kg)**
Starting	711,743,169	4,123,270,526	4,123,271	12.1%	5.793
Feeding	684,368,429	5,773,660,562	5,773,661	17.0%	8.436
Finishing	670,949,440	23,916,321,219	23,916,321	70.3%	35.645
Breeding	50,393,463	188,345,566	188,346	0.6%	3.738
Total		34,001,597,873	34,001,598	100%

**Table 6:** **Breakdown by swine antimicrobial combination**

**Starting Phase**						
**Antimicrobial(AM) combination**	**Total number of swine**	**Percent of swine treated**	**feed (lbs/day)**	**Avg days used**	**AM dose (g/lb)**	**Total in kg**
1. chlortetracycline sulfathiazole penicillin	711,743,169	20%	2	35	0.100	996,440
2. chlortetracycline sulfamethazine penicillin	711,743,169	20%	2	35	0.100	996,440
3. tylosin	711,743,169	40%	2	35	0.025	498,220
4. virginiamycin	711,743,169	4%	2	35	0.004	7,972
5. chlortetracycline	711,743,169	50%	2	35	0.040	996,440
6. oxytetracycline	711,743,169	40%	2	35	0.025	498,220
7. apramycin	711,743,169	10%	2	14	0.065	129,537
Total						4,123,271
**Feeding Phase**						
**Antimicrobial combination**	**Total number of swine**	**Percent of swine treated**	**feed (lbs/day)**	**Avg days used**	**AM dose (g/lb)**	**Total in kg**
1. chlortetracycline sulfathiazole penicillin	684,368,429	10%	4	38	0.1000	1,040,240
2. chlortetracycline sulfamethazine penicillin	684,368,429	7%	4	15	0.1000	287,435
3. tylosin sulfamethazine	684,368,429	5%	4	38	0.0900	468,108
4. carbadox	684,368,429	12%	4	38	0.0225	280,865
5. chlortetracycline	684,368,429	45%	4	38	0.0350	1,638,378
6. tylosin	684,368,429	30%	4	38	0.0175	546,126
7. bacitracin	684,368,429	55%	4	38	0.0150	858,198
8. virginiamycin	684,368,429	4%	4	38	0.0040	16,644
9. arsanilic acid	684,368,429	2%	4	38	0.0300	62,414
10. bambermycin	684,368,429	2%	4	38	0.0010	2,080
11. oxytetracycline	684,368,429	25%	4	38	0.0200	520,120
12. oleandomycin	684,368,429	2%	4	38	0.0040	8,322
13. lincomycin	684,368,429	4%	4	38	0.0080	33,288
14. efrotomycin	684,368,429	2%	4	38	0.0055	11,443
Total						5,773,661
**Finishing Phase**						
**Antibiotic combination**	**Total Number of swine**	**Percent of swine treated**	**feed (lbs/day)**	**Avg days used**	**AM dose (g/lb)**	**Total in kg**
1. chlortetracycline sulfathiazole penicillin	670,949,440	12%	6.2	86	0.1250	5,366,254
2. tylosin sulfamethazine	670,949,440	5%	6.2	72	0.1000	1,497,559
3. carbadox	670,949,440	15%	6.2	45	0.0250	701,981
4. chlortetracycline	670,949,440	55%	6.2	86	0.0350	6,886,692
5. tylosin	670,949,440	30%	6.2	86	0.0100	1,073,251
6. bacitracin	670,949,440	60%	6.2	86	0.0250	5,366,254
7. arsanilic acid	670,949,440	3%	6.2	86	0.0450	482,963
8. bambermycin	670,949,440	6%	6.2	86	0.0010	21,465
9. oxytetracycline	670,949,440	30%	6.2	86	0.0200	2,146,501
10. oleandomycin	670,949,440	5%	6.2	86	0.0056	100,617
11. efrotomycin	670,949,440	5%	6.2	86	0.0073	129,684
12. lincomycin	670,949,440	4%	6.2	86	0.0100	143,100
Total						23,916,321
**Breeding Phase**						
**Antimicrobial combination**	**Total number of swine**	**Percent of swine treated**	**feed (lbs/day)**	**Avg days used**	**AM dose (g/lb)**	**Total in kg**
1. chlortetracycline	50,393,463	85%	5	20	0.04	171,338
2. arsanilic acid	50,393,463	5%	5	20	0.045	11,339
3. bambermycin	50,393,463	25%	5	20	0.001	1,260
4. oxytetracycline	50,393,463	25%	5	14	0.005	4,409
Total						188,346

### By antibiotic class

In Tables 7 and 8 below, we calculate the quantities of antimicrobials used, by weight, from major antibiotic classes. Mellon et al. provided concentrations of each antimicrobial in each antimicrobial combination in Tables 4 and 6. We then followed the formulas and assumptions listed in the methods section to arrive at the totals by class as listed in Tables 7 and 8 below.

**Table 7:** **Antimicrobial use by class in poultry**

**Antibiotic Class**	**Total (kg)**
Tetracyclines	613,120
Penicillins	61,312
Macrolides	164,985
Coccidiostats*	3,407,220
Arsenicals*	2,879,624

* Many arsenical compounds function as coccidiostats.

**Table 8:** **Antimicrobial use by class in swine**

**Antibiotic Class**	**Total (kg)**
Sulfonamides	4,457,557
Tetracyclines	16,336,823
Penicillins	1,737,362
Macrolides	3,209,370
Aminoglycosides	129,537
Arsenicals	556,716

Table 7 indicates that coccidiostats and arsenicals are the most common antimicrobials used in poultry production by weight. Macrolides, penicillins, and tetracyclines are also used, with resulting implications for antimicrobial resistance and public health.

For swine, Table 8 indicates that tetracyclines, sulfonamides, macrolides, and penicillins are the largest antimicrobial classes by weight used in production. These classes of antimicrobials are also used frequently in human medicine.

## Discussion

The results of our calculations indicate that overall, 4.5 million kg [9.9 million lbs] of antimicrobials were used for poultry production and 34 million kg [75 million lbs] for swine production in China for 2012. These results use US data on food animal antimicrobial utilization with the assumption that Chinese methods are similar. Arsenicals account for 2.9 million kg [6.4 million lbs] in poultry production and only 556,000 kg [1.2 million lbs] in swine production. Calculations based on these two species alone account for 38.5 million kg [84.9 million lbs] of antimicrobials. Additionally, our methodology does not include all types of meat production (notably all avians), and thus total antimicrobial use, by weight, may be considerably larger for aggregate food animal production. This is important to note given China also produces large quantities of duck: 2,205,926,000 birds for 2012 [18]. Furthermore, as annual production and consumption increases, this model would predict that increasing volumes of antimicrobials would also be used each year [18].

A comparison to US antimicrobial consumption is useful. It is argued that the current US consumption of antimicrobials in both human medicine and animal feeds is too high, unnecessary, and harmful [35,45,46]. The total use of antimicrobials by weight for 2011 was 17 million kg [37.6 million lbs] [41,47]. Of this total, animal production accounted for 13.6 million kg [29.9 million lbs]. Our estimates of antimicrobial use in swine and poultry production in China are 38.5 million kg [84.9 million lbs]. This number is almost three times the amounts used for food animal production in the United States. However, it should be noted that this number is consistent with the higher production of swine in China [18].

The data on which we based our calculations has limitations. First, we do not have information on the extent to which food animal production in China is similar to that in the US. However, we have evidence that the shift from small farms to larger animal feeding operations is happening at a rapid pace, as discussed in the introduction. In addition, we do not have exact knowledge of the antibiotic regimens used in Chinese poultry and swine production. The lack of a monitoring and regulatory framework makes identification of such regimens difficult in China [36]. Moreover, we do not know how farm sizes may alter the use of antibiotics in feeds on Chinese farms. Given that industrial scale farming has increasing market share, we felt it was acceptable to generalize from the Mellon et al. model.

Furthermore, we assumed that animal feeds in 2014 Chinese food animal production were similar to the Mellon et al model. Antimicrobial usage in animal feeds can change. For example, in 2005, the FDA began restricting fluoroquinolone and later, arsenical use in poultry feeds [48-52]. Additionally, not all antimicrobials that are used in animal feeds were included in the original analysis by Mellon et al. Notably, they failed to include quinolones (enrofloxacin, norofloxacin, ofloxacin), which are clinically important drugs that monitoring systems continue to detect in poultry feeds [53,54]. Moreover, in China, multiple studies have shown residual antimicrobials in livestock manure including the presence of quinolones [55-57]. While the FDA regulates American livestock antimicrobial use to an extent, we are not aware of such regulation in China. Direct comparisons of residual antimicrobial masses in manure and the environment between the US and China are not available. Finally, quinupristin/dalfopristin was not included in these estimates, and it is known to be used in US poultry feeds. Hence, these agents should be accounted for in future research.

## Conclusions

In this paper, we estimated the quantity of antimicrobials used in Chinese swine and poultry production at 38.5 million kg [84.9 million lbs]. We anticipate challenges to this number and have outlined limitations in the data and methods above, notably the application of US antimicrobial utilization estimates to China. As with the estimate by Mellon et al. in the US, we hope this paper will stimulate discussion and collection of information on antimicrobial utilization in animal feeds in China. Ultimately, better information is needed to reach and ensure sound policy decisions on these practices.

## Abbreviations

CAFO: Concentrated animal feeding operation
FDA: Food and drug administration
FAO: Food and agriculture organization
FAOSTAT: Statistics division of the food and agriculture organization
